# MAL gene overexpression as a marker of high-grade serous ovarian carcinoma stem-like cells that predicts chemoresistance and poor prognosis

**DOI:** 10.1186/s12885-017-3334-1

**Published:** 2017-05-25

**Authors:** Laura Zanotti, Chiara Romani, Laura Tassone, Paola Todeschini, Renata Alessandra Tassi, Elisabetta Bandiera, Giovanna Damia, Francesca Ricci, Laura Ardighieri, Stefano Calza, Sergio Marchini, Luca Beltrame, Germana Tognon, Maurizio D’Incalci, Sergio Pecorelli, Enrico Sartori, Franco Odicino, Antonella Ravaggi, Eliana Bignotti

**Affiliations:** 1 “Angelo Nocivelli” Institute of Molecular Medicine, Division of Obstetrics and Gynecology, University of Brescia, P.le Spedali Civili 1, 25123 Brescia, Italy; 2 Department of Oncology, IRCCS, “Mario Negri” Institute for Pharmacological Research, Milan, Italy; 3 Department of Pathology, ASST Spedali Civili di Brescia, Brescia, Italy; 4 Department of Molecular and Translational Medicine, University of Brescia, Brescia, Italy; 5 Department of Medical Epidemiology and Biostatistics, Karolinska Institutet, Stockholm, Sweden; 6 Division of Obstetrics and Gynecology, ASST Spedali Civili di Brescia, Brescia, Italy; 7 Department of Obstetrics and Gynecology, University of Brescia, Brescia, Italy

**Keywords:** Cancer stem cells, Epithelial ovarian cancer, Chemoresistance, MAL

## Abstract

**Background:**

The existence of cancer stem cells (CSCs) within a tumor bulk has been demonstrated for many solid tumors including epithelial ovarian carcinoma (EOC). CSCs have been associated to tumor invasion, metastasis and development of chemoresistant recurrences. In this context, we aim to characterize EOC CSCs from the molecular point of view in order to identify potential biomarkers associated with chemoresistance.

**Methods:**

We isolated a population of cells with stem-like characteristics (OVA-BS4 spheroids) from a primary human EOC cell line under selective conditions. OVA-BS4 spheroids were characterized for drug response by cytotoxicity assays and their molecular profile was investigated by microarray and RT-qPCR. Finally, we performed a gene expression study in a cohort of 74 high-grade serous EOC (HGSOC) patients by RT-qPCR.

**Results:**

Spheroids exhibited properties of self-renewal and a pronounced expression of well-known stem cell genes. Moreover, they demonstrated greater resistance towards several anticancer drugs compared to parent cell line, consistent with their higher ABCG2 gene expression. From microarray studies MAL (T-cell differentiation protein) emerged as the most up-regulated gene in spheroids, compared to parent cell line. In HGSOC patients, MAL was significantly overexpressed in platinum-resistant compared to platinum-sensitive patients and resulted as an independent prognostic marker of survival.

**Conclusions:**

This investigation provides an important contribution to the identification of molecular markers of ovarian CSCs and chemoresistance. Successful translation of molecular findings would lead to a better comprehension of the mechanisms triggering chemoresistant recurrences, to the individuation of novel therapeutic targets and to the personalization of treatment regimens.

**Electronic supplementary material:**

The online version of this article (doi:10.1186/s12885-017-3334-1) contains supplementary material, which is available to authorized users.

## Background

Epithelial ovarian carcinoma (EOC) is the most lethal gynecological malignancy, with high-grade serous carcinoma (HGSOC) being the archetypical EOC, responsible for the highest number of cases and fatality rate. Despite improvements in debulking surgery and initial good response to platinum-based chemotherapy, overall survival for EOC patients remains poor with a 5-year survival rate virtually unchanged for the past 30 years [1]. One of the most important causes of failure in EOC treatment is the development of resistance to paclitaxel- and platinum-based chemotherapy [2]. Indeed, almost 80% of patients, who initially respond to adjuvant chemotherapy, subsequently experience relapse, typically less responsive to current chemotherapy strategies, and die for disease progression. Thus, the identification of molecular markers related to EOC chemoresistance is of crucial importance, because they may represent suitable targets for new therapeutic approaches [3].

One emerging model for the development of drug-resistant tumors invokes a pool of self-renewing malignant progenitors, known as cancer stem cells (CSCs) [4]. CSCs have been originally identified in leukemia [5] and more recently described in solid tumors, such as breast [6], colon [7] and ovarian carcinoma [8, 9].

According to this hypothesis, ovarian CSCs have been defined as a rare subpopulation of cells within a heterogeneous ovarian tumor, capable of forming and sustaining tumor growth, being characterized by the ability to self-renew, as well as the possibility to terminally differentiate.

The fundamental property of CSCs is their resistance to both chemotherapy and radiation. For this reason, CSCs represent a small proportion of cells within the tumor bulk, which potentially survives conventional treatments, becoming the putative mediators of recurrent disease and tumor progression [3, 10]. Consequently, there is a strong interest to identify this cell population and to functionally characterize its pathobiology, since CSCs may represent an important target to develop new therapeutic strategies.

The establishment of long-term cultures of cells with stem-like characteristics represents a step of crucial importance, providing a suitable model to study CSCs in vitro*.*

In this regard, recent studies have focused on the isolation and characterization of stem-like cells derived from human EOC cell lines [11, 12].

In this study, we firstly isolated a population of cells with stem-like characteristics from a primary HGSOC cell line and then we characterized its gene expression profile by microarrays. Based on our results, MAL emerged as the top up-regulated gene in stem-like chemoresistant cells, so we tested its expression in a cohort of HGSOC tissues with the aim to find a correlation with chemoresistance and prognosis.

## Methods

### Cell culture and tumor spheroid assay

The primary EOC cell line OVA-BS4 was established after sterile processing of a surgical biopsy from a metastatic tumor of high-grade serous histotype, as previously described [13]. The cell line (hereafter called parent OVA-BS4) was maintained in RPMI supplemented with 10% FBS, with an antibiotic-antimycotic solution in a humidified 5% CO_2_ incubator at 37 °C. Parent OVA-BS4 was evaluated by immunocytochemical staining with antibody against pan-cytokeratin to check epithelial purity.

OVA-BS4 spheroids were isolated from parent OVA-BS4 cell line grown under selective culture conditions. In detail, parent OVA-BS4 cells were trypsinized and placed at a density of 5 × 10^5^/ml in ultra-low attachment plates (Corning, New York, USA), in serum-free DMEM/F12 medium (Gibco, Life Technologies, Carlsbad, California, USA) supplemented with 5 μg/ml human insulin (Sigma, St. Louis, Missouri, USA), 20 ng/ml human recombinant epidermal growth factor (EGF, Gibco), 10 ng/ml human recombinant basic fibroblast growth factor (bFGF, Gibco) and B27 Supplement (Gibco) [9]. The culture medium was replaced twice a week, by centrifuging at 500 rpm for 5 min to remove dead cell debris. Weekly, non-adherent spheroids, potentially enriched in stem-like cells, were mechanically and enzymatically dissociated by incubation in a trypsin solution for 3 min at 37 °C and then re-seeded in the same culture conditions.

Both parent OVA-BS4 cell line and OVA-BS4 spheroids were authenticated by short tandem repeat (STR) DNA profiling. STR profiling was performed using PowerPlex® Fusion System (Promega, Madison, Wisconsin, USA) according to the manufacturer’s specifications and STR profiles were analyzed by GeneMapper 3.2.1 software.

### Patients’ samples

The present study was performed following the Declaration of Helsinki set of principles and approved by the Research Review Board -the Ethic Committee- of the ASST Spedali Civili, Brescia, Italy (study reference number: NP1676). Written informed consent was obtained from all patients enrolled. A total of 74 snap-frozen HGSOC biopsies were obtained from the Division of Obstetrics and Gynecology, ASST Spedali Civili, University of Brescia, Italy, between June 2002 and September 2013. All patients underwent a radical surgical tumor debulking and a complete staging procedure, including total abdominal hysterectomy, bilateral salpingo-oophorectomy, omentectomy and pelvic and periaortic lymph node sampling, with cytological evaluation of ascites or peritoneal washings.

Tumor tissues were sharp-dissected and snap-frozen in liquid nitrogen within 30 min after surgery and stored at −80 °C until further processing. For each sample, a specular hematoxylin-eosin section was reviewed by a pathologist and only samples containing at least 70% of tumor epithelial cells were used for the following experiments.

Patients with a past or concomitant history of malignancy were excluded from the study. No patient received chemotherapy before surgery, while all patients underwent adjuvant platinum-based therapy after surgery. Age, residual tumor after surgery (RT), treatment regimen and survival parameters were recorded by chart review for each patient. All patients presented advanced-stage disease (FIGO stage III-IV) and were divided in platinum-resistant (*n* = 39), with platinum-free interval (PFI) <6 months and platinum-sensitive (*n* = 35), with PFI >12 months*.* The PFI was defined as the last date of platinum dose until progressive disease is documented [14]. The clinicopathological characteristics of 74 HGSOC patients are summarized in Table 1.

**Table 1:** Clinicopathological characteristics of 74 HGSOC patients

	HGSOC patients	
	platinum-resistant	platinum-sensitive
	*n = 39*	*n = 35*
age at diagnosis (years); mean (range)	66 (36–82)	57 (42–80)
Residual Tumor after surgery; *n*		
RT = 0 cm	0	12
RT > 0 cm	39	23

For survival analysis, patients were followed from the date of surgery until death or for at least two years. Progression free survival (PFS) was considered as time interval from surgery to the first appearance of disease recurrence/progression after treatment, while overall survival (OS) was defined as the time interval from diagnosis to the date of death due to cancer, or the last observation.

### PKH-26 labeling

OVA-BS4 spheroids were dissociated mechanically and by EDTA treatment, and single cells were stained with 1 μM PKH-26 dye (Sigma) for 3 min according to manufacturer’s instructions, and plated at low density in low adherence 6-well plates. Fluorescent images were collected using a fluorescence microscope Axiovert 200.

### Phenotypic characterization by cytofluorimetric analysis

Parent OVA-BS4 and OVA-BS4 spheroids were dissociated mechanically and by EDTA treatment. Cell suspensions were counted, washed twice with PBS, and distributed at 200,000 cells per tube. Flow cytometry analysis was performed with the monoclonal antibodies: CD24 (FITC mouse Anti-Human CD24, clone ML5, BD PharmingenTM), CD44 (PE Mouse anti-human CD44, clone G44–26, BD PharmingenTM), CD117 (PE-CyTM5 mouse anti-human CD117, clone YB5.B8, BD PharmingenTM), CD133 (PE Mouse anti-human CD133/2, clone AC141, Miltenyi Biotec GmbH, Bergisch Gladbach, Germany). FITC Mouse IgG2a K (clone G155–178, BD PharmingenTM), PE mouse IgG2b K (clone27–35, BD PharmingenTM), PE-CyTM5 mouse IgG1 k (clone MOPC-21, BD PharmingenTM) and PE mouse IgG1 k (clone MOPC-21, BD PharmingenTM) were used as isotype controls. Mouse monoclonal antibodies were diluted and incubated according to the manufacturer’s instructions. Cells were acquired on a FACS-Calibur flow cytometer and samples were analyzed by Cell Quest Pro Software (BD Biosciences).

### Drug cytotoxicity assays

Parent OVA-BS4 and OVA-BS4 spheroids were dissociated by trypsin and seeded at the concentration of 1.7 × 10^5^ cells/ml onto 96-well plates, according to the specific culture conditions.

After 72 h, exponentially growing cells were treated with different doses of six anticancer agents: cisplatin (DDP; Sigma), paclitaxel (PTX; ChemieTek, Indianapolis, USA), etoposide (VP16; Sigma), PS341 (Selleckchem, Houston, USA), doxorubicin (DOXO; Sigma) and trabectedin (ET; PharmaMar, Madrid, Spain).

Each condition was set up in five replicates and three independent experiments were performed.

After 96 h from treatment, cell viability was monitored by MTS assay (CellTiter® 96 AQueous One Solution Cell Proliferation Assay; Promega) and optical density reading at 490 nm. The control group was represented by untreated cells. Cell viability percentage was calculated using the formula = (mean absorbance of the test well/mean absorbance of the control) × 100. Half-maximal inhibitory concentration (IC50) was calculated for each drug.

### Total RNA extraction

Total RNA was extracted from parent OVA-BS4 and OVA-BS4 spheroids using All Prep DNA/RNA/miRNA Universal kit (Qiagen, Valencia, CA, USA), according to manufacturer’s instructions.

Total RNA was extracted from tissue samples using TRIzol® Reagent (Life Technologies, Carlsbad, California, USA), followed by a purification with RNeasy MinElute Cleanup® kit (Qiagen), according to manufacturer’s instructions.

RNA concentration and 260/280 absorbance ratio (A_260/280_) were measured with Infinite M200 spectrophotometer (Tecan, Männedorf, Switzerland), while RNA integrity was assessed with RNA 6000 Nano LabChip kit using the Agilent 2100 Bioanalyzer (Agilent Technologies, Palo Alto, CA, USA). RNA integrity number (RIN), generated with Agilent 2100 Expert software, was superior to 8 for all RNA samples.

### Microarray analysis

Microarray experiments were performed on parent OVA-BS4 and OVA-BS4 spheroids using the commercially available G4851B human whole GE Microarray kit (SurePrint G3 Human Gene Expression 8 × 60 K v2 Microarray Kit Agilent Technologies) according to manufacturer’s instructions. Fluorescence intensities were measured by Feature Extraction software v11 (Agilent Technologies). Raw data were pre-processed, removing features marked as unreliable by the scanning software in at least 60% of the samples, and afterward normalized using the “quantile” method. Differential analysis was carried out with linear models for microarray analysis [15], correcting the resulting *p*-values for multiple testing with the False Discovery Rate (FDR) method [16]. Only genes with a corrected *p*-value of less than 0.01 and regulated at least two fold compared to controls were called significant. In accordance to the MIAME guidelines, raw and processed data have been submitted to the Array Express repository (ID pending).

Gene Enrichment Analysis for selected genes was performed based on a cancer stem cell (CSC)-specific pathway gene list (Additional file 1: Table S1) and a 34 gene-based signature predictive of chemoresistance (Additional file 2: Table S2), using Fisher Exact Test. HUGO gene symbols were used as matching criteria after duplicates removal.

### Quantitative real-time PCR

One microgram of RNA from parent OVA-BS4 and OVA-BS4 spheroids was reverse-transcribed using random hexamers according to the SuperScript TM II reverse transcriptase protocol (Life Technologies).

Quantitative Real-Time PCR (RT-qPCR) was performed on the ABI PRISM 7000 Sequence detection System (Life Technologies, Applied Biosystems, Applera UK, Cheshire, UK) using the TaqMan Universal PCR master mix and the following assays (Life Technologies): Hs01053790_m1 (ABCG2), Hs00180254_m1 (ALDH1A1), Hs01030099_m1 (CCNB1), Hs00765553_m1 (CCND1), Hs00265816_s1 (CLDN3), Hs00533616_s1 (CLDN4), Hs04260366_g1 (NANOG), Hs01062014_m1 (NOTCH1), Hs00195591_m1 (SNAI1), Hs00415716_m1 (SOX2), Hs00185584_m1 (VIM), Hs00232783_m1 (ZEB1), Hs00242748_m1 (MAL).

Reaction and thermal cycling conditions were performed as previously reported [17].

The comparative threshold cycle (Ct) method was used to determine Fold Changes (FC) in gene expression in each sample, normalized using the geometric mean of three reference genes, GAPDH (Hs99999905_m1), GUSB (Hs00939627_m1) and HPRT1 (Hs02800695_m1). All experiments were performed in triplicate.

Moreover, cDNA obtained from 74 snap-frozen HGSOC biopsies were evaluated for the expression of MAL gene by RT-qPCR, using the following assay: Hs00242748_m1 (Life Technologies). MAL gene expression levels were normalized using HPRT1 (Hs02800695_m1; Life Technologies), as the most stable reference gene among the four genes tested (HPRT1, TBP, PPIA, GAPDH).

### Statistical analysis

Robust linear model was used to compare log-transformed IC50 values in OVA-BS4 spheroids towards parent OVA-BS4 in drug cytotoxicity assays. The correlation between microarray and RT-qPCR data for MAL gene expression was evaluated using Spearman rank correlation. The variations in RT-qPCR gene expression between parent OVA-BS4 and OVA-BS4 spheroids, as well as between platinum-resistant and platinum-sensitive patients, were evaluated by a t-test.

Survival models were fitted using Cox proportional hazard models, while survival curves were drawn based on the Kaplan-Meier methods.

The impact of MAL expression on prognosis was evaluated categorizing the RT-qPCR values in tertiles computed on the whole cohort. In all analyses, the significance level was 5%. All analyses were performed using R (version 3.3.0).

## Results

### Short tandem repeat (STR) analysis

Comparison of DNA fingerprinting results in DSMZ database confirmed the unique identity of both OVA-BS4 cell lines (parental and spheroids). The complete STR profile is shown in Additional file 3: Table S3.

### Self-renewing spheroids could be successfully isolated from a primary EOC cell line

**Fig. 1:**
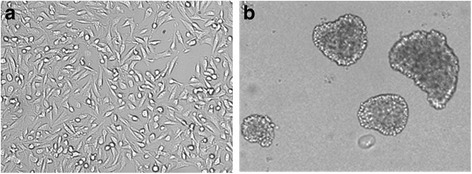
Morphology of parent OVA-BS4 (magnification 200X) (**a**) and OVA-BS4 spheroids (magnification 100X) (**b**). Parent OVA-BS4 were cultured in RPMI 10%FBS in adhesion condition, whereas OVA-BS4 spheroids were obtained from parent OVA-BS4 cell line under selective culture condition (serum-free DMEM/F12 medium supplemented with 5 μg/ml human insulin, 20 ng/ml EGF, 10 ng/ml bFGF, and B27 Supplement, in ultra low-attachment plates)

Under selective culture conditions OVA-BS4 primary cell line aggregated into floating spheroid clusters (Fig. 1). When mechanically or enzymatically dissociated, spheroids generated single cells that spontaneously re-aggregates into secondary spheroids under the same selective conditions. This procedure has been repeated weekly for over 80 passages, without appreciable changes in cell shape. After withdrawal of growth factors and addition of 10% FBS, single cells collected from dissociated spheroids showed adhesion to the plastic substrates and recovery of their epithelial morphology, surviving subsequent passages.

**Fig. 2:**
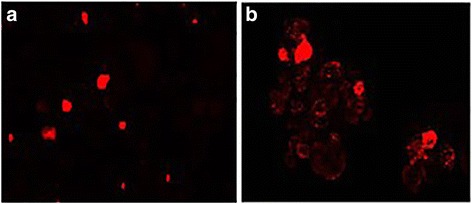
Images of PKH-26 staining during spheroid formation (magnification 100X). Single cells labeled with PKH-26, derived from mechanic dissociation of OVA-BS4 spheroids; day 0 (**a**). After 4 days, most of the cells underwent PKH-26 dilution in the newly formed spheroids while few retained the red label (**b**)

Self-renewal ability of spheroid-forming cells, reported as one of the main property of CSCs, was investigated by staining with PKH-26, a lipophilic vital fluorescent dye that irreversibly binds to cell membranes and undergoes a dilution in daughter cells during cellular replication. In particular, this method can be applied to the study of CSCs, allowing discriminating terminally differentiated cells from cells with a potential of stemness. Cells derived from disaggregated OVA-BS4 spheroids were incubated with PKH-26 and the formation of spheroids was followed using a fluorescence microscope. As shown in Fig. 2a, at the day of plating (T0) every cell was fluorescent and appeared as a single red-stained cell. After 4 days of culture, a clear red-stained cell was visible in the center of the newly formed spheroids, whilst the other cells were less positive for the PHK-26 label (Fig. 2b). The same finding was observed after 7 days of culture. This result suggested that OVA-BS4 spheroids were not mere aggregates of cells, but likely the result of the clonal expansion of single PHK-26 positive cells, which underwent an asymmetrical division giving rise to a quiescent daughter cell as well as daughter cells capable of consecutive divisions, thus causing total dye quenching.

### Self-renewing spheroids are characterized by significantly higher surface expression of CD133 compared to parental cell line

**Fig. 3:**
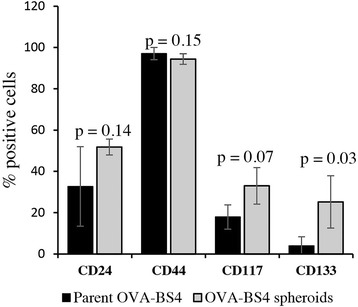
Expression pattern of CSC surface markers in adherent cell lines and spheroids by flow cytometric analysis. The expression is represented as a percentage of positive cells and is derived from at least three independent experiments. Data represent mean ± SD. Student’s t-test

To evaluate the surface expression patterns of ovarian CSC markers, CD24, CD44, CD117 and CD133 expression was investigated in both spheroids and parent OVA-BS4 (Fig. 3). FACS analysis demonstrated that CD133 was significantly more expressed in spheroids compared to parent cell line. Mean ± DS of CD133 positive cells was 25 ± 13% and 4 ± 4%, respectively (*p* = 0.03). The percentage of CD24 (Mean ± DS: 52 ± 4% and 33 ± 13%, *p* = 0.14) and CD117 (Mean ± DS: 33 ± 9% and 18 ± 6%, *p* = 0.07) positive cells was higher in OVA-BS4 spheroids compared to parent OVA-BS4, but did not reach the statistical significance. Both cell lines displayed high levels of CD44 (mean ± DS = 94 ± 4% in spheroids and 97 ± 4% in adherent cells, *p* = 0.15).

### Molecular characterization of OVA-BS4 spheroids reveals a stem-like phenotype

To investigate the molecular phenotype of OVA-BS4 spheroids, the gene expression profile was analyzed by Agilent human cDNA microarray analysis. From the comparison between spheroids and parent cell line, 6964 differentially expressed genes were identified: 3677 were up-regulated and 3287 down-regulated. The list of the up-regulated and down-regulated genes was reported in Additional file 4.

Out of the 6964 significant genes, we identified 4381 unique symbols (out of a total of 36,475 unique symbols in the chip). To perform a gene set enrichment analysis, a set of 84 unique genes involved in CSC-related pathways were considered. Using a Fisher Exact Test, we found a significant enrichment (OR = 3.68, *p* < 0.0001) with a total of 28 (33.3%) selected genes included in CSC-related pathways, where 10.1 (12.0%) genes were expected by chance only (Additional file 1: Table S1). Moreover, using the same statistical approach, we found a significant enrichment (OR = 3.06, *p* < 0.0053) with a total of 10 out of 34 selected genes (29.4%) included in chemoresistance-associated gene signature (Additional file 2: Table S2).

**Fig. 4:**
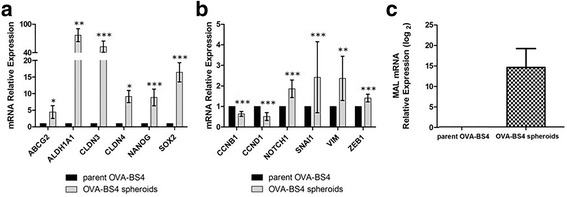
**a**, **b** Expression of selected genes mRNAs associated with stemness and epithelial to mesenchymal transition in parent OVA-BS4 and OVA-BS4 spheroids. Data are displayed as mean ± SE of three independent experiments and are expressed as relative expression ratios using parent OVA-BS4 as a reference. Genes are divided into two graphs based on their relative expression. *p < 0.05; **p < 0.01; ***p < 0.001. **c** MAL mRNA expression by RT-qPCR in parent OVA-BS4 and OVA-BS4 spheroids. Data are displayed as mean ± SE of nine independent experiments and are expressed as relative expression in logarithmic scale using parent OVA-BS4 as a reference

Furthermore, we investigated by RT-qPCR the expression of 12 genes known to be correlated with stemness, epithelial to mesenchymal transition (EMT) and multidrug resistance. Gene expression levels are displayed in Fig. 4a, b. The expression of putative stem cell markers such as ALDH1A1, NANOG, NOTCH1, SOX2 were significantly higher in OVA-BS4 spheroids compared to parent OVA-BS4 (*p* = 0.002 for ALDH1A1; *p* < 0.001 for the others). The gene expression of ABCG2, a membrane efflux transporter implicated in chemotherapy resistance, was significantly over-expressed in OVA-BS4 spheroids (*p* = 0.04). Moreover, OVA-BS4 spheroids revealed a higher expression of SNAI1 (*p* < 0.001), VIM (*p* = 0.002) and ZEB1 (*p* < 0.001), implicated in EMT process, and an elevated expression of tight junctions CLDN3 (*p* < 0.001) and CLDN4 genes (*p* = 0.01) compared to parent OVA-BS4. Finally, OVA-BS4 spheroids displayed a significant downregulation of CCNB1 and CCND1 (both *p* < 0.001) compared to parent OVA-BS4, as indication of their slow cycling phenotype and as a possible manifestation of dormancy and quiescence typical of CSCs.

### OVA-BS4 spheroids display resistance to traditional chemotherapeutics

**Fig. 5:**
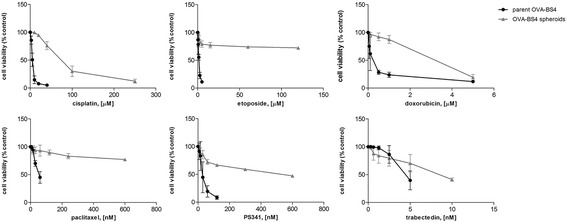
OVA-BS4 spheroids are highly resistant to conventional chemotherapeutics. Parent OVA-BS4 and OVA-BS4 spheroids were treated for 96 h with increasing concentrations of the indicated drugs, and proliferation rates were estimated by MTS assay. Dose-response curves for parent OVA-BS4 (*bold line*) and OVA-BS4 spheroids (*grey line*) indicate the percentage of cell viability compared to untreated control and are represented as mean ± SD of at least three independent experiments

To characterize the pharmacological profile and to determine drug sensitivities of parent OVA-BS4 and OVA-BS4 spheroids, a comparative assay was performed using anticancer agents with different mechanisms of action. In particular, we evaluated drugs commonly used in the treatment of EOC (i.e. DDP, PTX, VP16, DOXO, ET), as well as a new cytotoxic targeted agent, the proteasome inhibitor PS341. To this aim, parent OVA-BS4 and OVA-BS4 spheroids were incubated for 4 days with the drugs at different concentrations and comparative dose-response curves, representing the relative viabilities after treatment, were constructed. As shown in Fig. 5, OVA-BS4 spheroids demonstrated substantial higher viability compared to parent OVA-BS4, indicative of higher resistance to all the anticancer drugs tested. The estimated IC50 mean values for all drugs are shown in Table 2.

**Table 2:** Estimated IC_50_ mean values for parent OVA-BS4 and OVA-BS4 spheroids

Drugs	parent OVA-BS4	OVA-BS4 spheroids	Fold change	*P* value
Cisplatin (μM)	5.72 ± 0.90	85.23 ± 60.10	14.9	<0.0001
Paclitaxel (nM)	62.09 ± 16.19	600.00^a^	>10	<0.0001
Etoposide (μM)	1.57 ± 0.18	120.00^a^	>76	<0.0001
Doxorubicin (μM)	0.22 ± 0.10	3.11 ± 0.29	14.1	<0.0001
PS341 (nM)	29.51 ± 11.70	530.83 ± 109.33	18.0	<0.0001
Trabectedin (nM)	5.05 ± 1.44	8.75 ± 0.35	1.7	0.002

^a^The value corresponds to the maximum dose tested, even if not sufficient to kill the 50% of cells

Stem-like OVA-BS4 spheroids showed significantly higher IC50 values for all the anticancer agents compared to parent OVA-BS4 (*p* = 0.002 for trabectedin; *p* < 0.0001 for the other drugs).

### MAL is a putative CSC marker associated with chemoresistance and survival

Among differentially expressed genes, we focused our attention on MAL, the top up-regulated gene in spheroids compared to adherent cell line (Fold Change =11.9; Additional file 4). RT-qPCR was used to validate microarray data regarding MAL gene in nine biological independent replicates. RT-qPCR results significantly correlated to the microarray data (Spearman’s coefficient = 0.99), confirming the significantly higher MAL gene expression in OVA-BS4 spheroids compared to parent OVA-BS4 (*p* < 0.001) (Fig. 4c).

To assess the predictive and prognostic potential of MAL gene, its expression was investigated by RT-qPCR on a homogenous cohort of advanced-stage HGSOC patients, classified according to their sensitivity to platinum-based treatment. The comparison between the group of 35 platinum-sensitive and 39 platinum-resistant patients demonstrated that MAL was significantly over-expressed in platinum-resistant HGSOC patients (Fold Change = 2.27; *p* = 0.011) and showed a significant association with the presence of residual tumor after surgery (RT) (Fold Change = 3.42; *p* = 0.0053).

As expected, RT, a known EOC clinical prognostic factor, showed a statistically significant association with OS (*p* = 0.006) and PFS (*p* < 0.001) in univariate survival analysis (Table 3).

**Table 3:** Univariate and multivariate analysis of overall survival (OS) and progression free survival (PFS) in relation to Residual Tumor after surgery and MAL mRNA expression. Model adjusted by age on continuous scale

Variables	OS				PFS			
	*n*	HR	95% CI	*p value*	*n*	HR	95% CI	*p value*
Univariate survival analysis								
MAL mRNA RQ (medium/high vs low tertile)	74	2.28	1.23–4.23	0.009	74	1.81	1.05–3.12	0.033
Residual Tumor after surgery (RT > 0 cm vs RT = 0 cm)	74	5.11	1.59–16.40	0.006	74	4.25	1.81–9.94	<0.001
Multivariate survival analysis								
MAL mRNA RQ (medium/high vs low tertile)	74	2.01	1.07–3.78	0.029	74	1.66	0.95–2.90	0.074
Residual Tumor after surgery (RT > 0 cm vs RT = 0 cm)	74	4.32	1.34–13.96	0.014	74	3.88	1.64–9.15	0.002

**Fig. 6:**
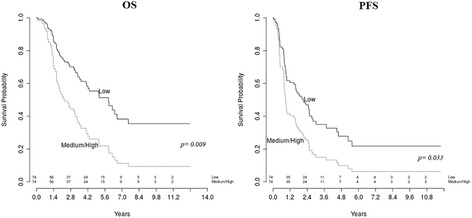
Kaplan-Meier survival curves for HGSOC patients according to MAL mRNA expression. Higher MAL mRNA levels (medium/high versus low tertiles) showed a significant association with short overall survival (OS) and poor progression free survival (PFS)

In addition, as displayed in Fig. 6, higher MAL mRNA levels were significantly associated with shorter OS (*p* = 0.009) and poor PFS (*p* = 0.033).

Age, RT and MAL mRNA levels were then included in a multivariate survival analysis. As shown in Table 3, high MAL mRNA expression and RT were identified as independent predictors for shorter OS. Moreover, RT maintained its role as an independent prognostic factor for poor PFS (*p* = 0.002), while higher MAL levels exhibited a trend towards significance (*p* = 0.074).

## Discussion

HGSOC displays the highest mortality rate of all gynecological cancers, showing early recurrence due to the development of chemoresistant disease.

CSCs, a small subpopulation of cells able to repopulate the tumor after chemotherapeutic treatments, are thought to contribute to the onset of chemoresistant recurrences in EOC.

Ovarian CSCs can be isolated from ascites and from primary or metastatic tumor specimens [8, 18]. In addition, long-term cancer cell cultures could maintain a cellular hierarchy, containing rare stem-like cells, progenitors and cells at different stages of differentiation, as already demonstrated for several human carcinoma cell lines [19].

Based on this assumption, in this paper we described the enrichment in stem-like cells, namely OVA-BS4 spheroids, starting from the parent primary EOC cell line OVA-BS4, previously established in our laboratory. OVA-BS4 spheroids were obtained using the tumor spheroid assay, a well-known method to examine the capacity of tumor cells to grow as multicellular spheroids under non-differentiating and non-adherent conditions [3]. After labeling with the fluorescent vital dye PKH-26, we observed that each tumor spheroid arose from a single cell demonstrating its ability of self-renewal, a typical property of CSCs.

Several studies have prospectively isolated CSCs by looking for the presence of extracellular markers that are thought to be CSC specific. Currently, while markers such as CD24, CD44, CD117 and CD133 have been frequently exploited to enrich for putative CSCs, no consensus has emerged [20]. The phenotypic characterization revealed that OVA-BS4 spheroids exhibited a more pronounced positivity to the above mentioned markers compared to parent cell line, although only CD133 and CD117 reached a statistical significance. Based on our findings, some of the surface markers investigated, in particular CD44, cannot be considered as reliable markers for defining a CSC population, since they do not characterize CSCs exclusively. Our data demonstrated that CD133 and CD117 can be considered as markers able to identify the population enriched in stem-like cells, as already demonstrated by other groups [21, 22].

The transcriptional profile of OVA-BS4 spheroids demonstrated high expression levels of several stemness-related genes, including NANOG and SOX2, two key transcription factors responsible for pluripotency induction and regulation of embryonic stem cells [23]. In addition, OVA-BS4 spheroids displayed elevated expression of NOTCH1 gene, whose signaling was reported to promote self-renewal of CSCs in several malignancies, regulating both the formation of CSCs and the acquisition of a mesenchymal phenotype, associated with drug resistance [24].

Moreover, OVA-BS4 spheroids exhibited a significant overexpression of ALDH1A1, an intracellular enzyme involved in different cellular functions, such as detoxification and endowed with a probable protective role against oxidative damage in stem cells, as reported in literature [25].

Compared to parent OVA-BS4, OVA-BS4 spheroids showed a higher expression of EMT-associated markers, like SNAI1, VIM and ZEB1, suggesting that they underwent biological changes characteristic of CSC enrichment. Indeed, EMT is a well-known process involved in tumor cell motility and tumor metastasis, as well as in the generation of CSCs [26].

Interestingly, OVA-BS4 spheroids revealed high expression of CLDN3 and CLDN4 genes. Several lines of evidence support the association of high CLDN4 expression with chemoresistance in ovarian cancer. A recent study [27] reported the overexpression of CLDN4 in platinum-resistant EOC patients compared to platinum-sensitive ones and demonstrated an increased sensitivity of ovarian cancer cells to cisplatin after in vitro suppression of CLDN4 expression by siRNA. Moreover, CLDN4 seemed to be involved in the regulation of spheroid formation, since knockdown of CLDN4 expression delayed spheroid formation in ovarian cancer cells [28]. The relevance of claudins in CSCs is beginning to emerge, thus further investigations aimed at elucidating their function in our CSCs model are warranted.

Like normal stem cells, OVA-BS4 spheroids showed a high expression of efflux transporters from the ABC gene family, which represents a mechanism to preserve more effectively their genome against chemical mutagens. In particular, we observed an elevated expression of ABCG2, a member of ABC transporter family, in OVA-BS4 spheroids compared to parent OVA-BS4. This drug transporter is associated with the acquisition of drug resistance in CSCs, since it is implicated in the active transport of genotoxic agents across cell membrane through ATP hydrolysis [29]. This hypothesis may be corroborated by our results obtained from the comparative drug cytotoxicity assays, which demonstrated a clear resistance of low-adherence OVA-BS4 spheroids towards all the compounds tested. Following this assumption, the overexpression of the ATP-binding cassette transporter ABCG2 may be a possible mechanism enabling low-adherence spheroids to escape the cytotoxic effects of chemotherapy, triggering an important mechanism of drug resistance [30]. ABCG2 is a broad-specificity drug transporter [31], since it can transport epipodophyllotoxins, like etoposide, and anthracyclines, like doxorubicin, as well as innovative tyrosine kinase inhibitors. The list of ABCG2 substrates is rapidly expanding, highlighting the wide involvement of this protein in chemoresistance mechanisms. Resistance to toxic agents is one of the most important biological characteristics of CSCs [10], suggesting their possible role in the determination of ovarian cancer recurrence after chemotherapy treatment.

Taking together, our data, based on the molecular and the pharmacological characterization of low-adherence OVA-BS4 spheroids, suggest that these cells possess intrinsic properties compatible with a CSCs phenotype. Our findings are strengthened by results from gene enrichment analysis on microarray data, which demonstrated OVA-BS4 spheroids enriched by genes associated with CSC properties and with characteristics of chemoresistance.

Since we demonstrated OVA-BS4 spheroids as a valuable in vitro model to study HGSOC chemoresistance, we investigated the expression of the gene MAL, the most up-regulated in OVA-BS4 spheroids compared to parent OVA-BS4, in a homogenous cohort of HGSOC patients. MAL was firstly discovered as a gene expressed during T-cell development, later it was found in polarized epithelial cells and localized in membrane microdomains suggesting a probable role in cell signaling [32]. The function of MAL in tumor cells is still controversial. In particular, a tumor suppressor activity was demonstrated in esophageal tumors [33], as well as in colorectal and gastric cancer [34, 35], while an intense MAL protein expression was detected in specific types of renal carcinoma and in thyroid follicular cell-derived carcinoma [36]. Moreover, MAL was found frequently hypermethylated in colon and breast cancer, thus explaining its reduced gene expression [37, 38]. Interestingly, MAL overexpression in cutaneous T-cell lymphoma was associated with resistance to alpha-interferon therapy [39], and its expression was indicative of poor prognosis in Hodgkin’s lymphoma [40]. In ovarian cancer, MAL expression was reported mostly in clear-cell and serous histotypes [41] and its increased expression was observed in short-term compared to long-term survivors [42]. Furthermore, elevated levels of MAL transcripts in ovarian cancer cell lines have been associated with resistance to cisplatin [43], indicating a possible implication of MAL in determination of in vitro platinum resistance.

Importantly, the present investigation demonstrated that MAL expression was correlated to platinum resistance in our cohort of HGSOC patients, confirming its involvement in chemoresistance. Moreover, in agreement with Berchuck [42], MAL gene emerged as an independent prognostic marker for HGSOC, being associated with shorter OS and PFS in multivariate survival analysis. To our knowledge, the present work represents the first report on the correlation between overexpression of MAL gene and EOC stem-like cells.

## Conclusion

In summary, we isolated and characterized the transcriptional pattern of a population enriched in stem-like cells that allowed the identification of MAL as a predictive marker of HGSOC chemoresistance and prognosis, potentially representing a new therapeutic target for platinum-resistant HGSOC. Further investigations about the transcriptional regulation of MAL and its functional role in platinum resistance mechanisms are warranted.

## Additional files

Additional file 1: Table S1. Genes related to cancer stem cells (CSC). List was previously compiled by SA biosciences (http://www.sabiosciences.com/rt_pcr_product/HTML/PAHS-176Z.html). Twenty-eight of these CSC-associated genes were among the 6964 significant genes differentially expressed between OVA-BS4 spheroids and parent cell line. (DOCX 13 kb)

Additional file 2: Table S2. Genes related to chemoresistance. A panel of 34 genes related to chemoresistant phenotype were derived from the literature. Ten of these genes were among the 6964 significant genes differentially expressed between OVA-BS4 spheroids and parent cell line. (DOCX 14 kb)

Additional file 3: Table S3. Short Tandem Repeat (STR) DNA profile of parent OVA-BS4 cell line and OVA-BS4 spheroids. (DOCX 12 kb)

Additional file 4: Up-regulated and down-regulated genes with a corrected *p*-value of less than 0.01 and expressed at least two fold higher and lower in OVA-BS4 spheroids compared to parent OVA-BS4. (XLS 1147 kb)

## Abbreviations

CSCs: Cancer stem cells
Ct: Comparative threshold cycle
EMT: Epithelial to mesenchymal transition
EOC: Epithelial ovarian carcinoma
FC: Fold changes
FDR: False discovery rate
HGSOC: High-grade serous ovarian carcinoma
IC50: Half-maximal inhibitory concentration
OS: Overall survival
PFI: Platinum-free interval
PFS: Progression free survival
RT: Residual tumor after surgery
RT-qPCR: Quantitative real-time PCR
STR: Short tandem repeat
