# Supplementary material for: MAL gene overexpression as a marker of high-grade serous ovarian carcinoma stem-like cells that predicts chemoresistance and poor prognosis
Source: BMC Cancer. 2017 May 25;17:366. doi: 10.1186/s12885-017-3334-1 (PMC5445497; doi:10.1186/s12885-017-3334-1)
Supplement: Supplementary file 1 — Genes related to cancer stem cells (CSC). List was previously compiled by SA biosciences (http://www.sabiosciences.com/rt_pcr_product/HTML/PAHS-176Z.html). Twenty-eight of these CSC-associated genes were among the 6964 significant genes differentially expressed between OVA-BS4 spheroids and parent cell line. (DOCX 13 kb) [file 12885_2017_3334_MOESM1_ESM.docx]

**ADDITIONAL FILE 1**

**Table S1**. Genes related to cancer stem cells (CSC). List was previously compiled by SA biosciences (<http://www.sabiosciences.com/rt_pcr_product/HTML/PAHS-176Z.html>). Twenty-eight (*) of these CSC-associated genes were among the 6964 significant genes differentially expressed between OVA-BS4 spheroids and parent cell line.

| **Functional Gene Grouping** | **HUGO gene symbols** |
| --- | --- |
| Cancer Stem Cell Markers | ABCB5, **ALCAM***, ALDH1A1, **ATXN1***, BMI1, CD24, CD34, CD38, CD44, ENG, ETFA, FLOT2, **GATA3***, **ITGA2***, **ITGA4***, ITGA6, **ITGB1***, KIT, MS4A1, **MUC1***, PECAM1, PROM1, PTPRC, THY1 |
| Proliferation | EGF, ERBB2, **KITLG***, LIN28B, **NOS2*** |
| Self-Renewal | **BMP7***, DNMT1, **FGFR2*** |
| Pluripotency | KLF4, LIN28A, MYC, **NANOG***, POU5F1, **SOX2*** |
| Asymmetric Division | **FOXP1***, HDAC1, MYCN, SIRT1, WNT1 |
| Migration and Metastasis | AXL, ID1, **IL8***, KLF17, **PLAT***, **PLAUR***, **SNAI1***, TWIST1, TWIST2, ZEB1, **ZEB2*** |
| Loss of Stemness | ALDH1A1, CD34, DACH1, FOXA2, PECAM1, PTCH1 |
| Signal Transduction Pathways: |  |
| Hippo Signaling | LATS1, **MERTK***, SAV1, TAZ, WWC1, **YAP1*** |
| Hedgehog Signaling | PTCH1, **SMO*** |
| Notch Signaling | **DLL1***, DLL4, JAG1, MAML1, NOTCH1, NOTCH2 |
| WNT Signaling | **DKK1***, EPCAM, FZD7, WNT1 |
| PI3K/AKT/mTOR Signaling | ABCG2, GSK3B |
| STAT/NFkB Signaling | **IKBKB***, JAK2, **NFKB1*** |
| Therapeutic Targets | ABCG2, ATM, AXL, **CHEK1***, DDR1, DKK1, EPCAM, FZD7, GSK3B, ID1, IKBKB, JAK2, KLF17, NFKB1, PTCH1, SMO, STAT3, **TGFBR1***, WEE1 |
