# Supplementary material for: MAL gene overexpression as a marker of high-grade serous ovarian carcinoma stem-like cells that predicts chemoresistance and poor prognosis
Source: BMC Cancer. 2017 May 25;17:366. doi: 10.1186/s12885-017-3334-1 (PMC5445497; doi:10.1186/s12885-017-3334-1)
Supplement: Supplementary file 2 — Genes related to chemoresistance. A panel of 34 genes related to chemoresistant phenotype were derived from the literature. Ten of these genes were among the 6964 significant genes differentially expressed between OVA-BS4 spheroids and parent cell line. (DOCX 14 kb) [file 12885_2017_3334_MOESM2_ESM.docx]

**ADDITIONAL FILE 2**

**Table S2** Genes related to chemoresistance. A panel of 34 genes related to chemoresistant phenotype were derived from the literature (1-6). Ten (*) of these genes were among the 6964 significant genes differentially expressed between OVA-BS4 spheroids and parent cell line.

| **Molecular Function** | **HUGO gene symbol** |
| --- | --- |
| ATP-binding cassette transporters | ABCC1, ABCC2, **ABCB1***, ABCG2 |
| ATP-ase copper transporters | **ATP7A***, **ATP7B***, CTR1 |
| Ribosomal proteins | RPL4, **RPL10***, RPL10A, RPL15, RPL18, **RPL28***, RPL29, RPS5, RPS19, RPS2, FAU, RPLP1 |
| Transcription factors | TFAP4, FOXM1 |
| Transcription regulators | **MACC1*** |
| Protein Kinase | EIF2AK2, **CDK2*** |
| Chromatine remodeling | **RBBP4*** |
| ER membrane receptors | SSR2 |
| Embryonic development | AES |
| Mitochondrial proteins | TUFM |
| Stress Resistance proteins | **HSPB1*** |
| Translation elongation factors | EEF1A1, EEF1G, EEF2 |
| Chemokine receptors | **CXCR4*** |
| Extracellular binding proteins | CYR61 |

**References**

1. Fletcher JI1, Haber M, Henderson MJ, Norris MD. ABC transporters in cancer: more than just drug efflux pumps. Nat Rev Cancer. 2010 Feb;10(2):147-56. doi: 10.1038/nrc2789. Epub 2010 Jan 15.

2. Li J, Jiang K, Qiu X, Li M, Hao Q, Wei L, Zhang W, Chen B1, Xin X. Overexpression of CXCR4 is significantly associated with cisplatin-based chemotherapy resistance and can be a prognostic factor in epithelial ovarian cancer. BMB Rep. 2014 Jan;47(1):33-8.

3. Zhang R, Shi H, Ren F, Li X, Zhang M, Feng W, Jia Y. Knockdown of MACC1 expression increases cisplatin sensitivity in cisplatin-resistant epithelial ovarian cancer cells. Oncol Rep. 2016 Apr;35(4):2466-72.

4. Shen H, Cai M, Zhao S, Wang H, Li M, Yao S, Jiang N. CYR61 overexpression associated with the development and poor prognosis of ovarian carcinoma. Med Oncol. 2014 Aug;31(8):117.

5. Zona S, Bella L, Burton MJ, Nestal de Moraes G, Lam EW. FOXM1: an emerging master regulator of DNA damage response and genotoxic agent resistance. Biochim Biophys Acta. 2014 Nov;1839(11):1316-22.

6. He DX, Xia YD, Gu XT, Jin J, Ma X. A transcription/translation-based gene signature predicts resistance to chemotherapy in breast cancer. J Pharm Biomed Anal. 2015 Jan;102:500-8. doi: 10.1016/j.jpba.2014.10.018. Epub 2014 Oct 27.
