# Supplementary material for: MAL gene overexpression as a marker of high-grade serous ovarian carcinoma stem-like cells that predicts chemoresistance and poor prognosis
Source: BMC Cancer. 2017 May 25;17:366. doi: 10.1186/s12885-017-3334-1 (PMC5445497; doi:10.1186/s12885-017-3334-1)
Supplement: Supplementary file 3 — Short Tandem Repeat (STR) DNA profile of parent OVA-BS4 cell line and OVA-BS4 spheroids. (DOCX 12 kb) [file 12885_2017_3334_MOESM3_ESM.docx]

**ADDITIONAL FILE 3**

**Table S3** Short Tandem Repeat (STR) DNA profile of parent OVA-BS4 cell line and OVA-BS4 spheroids.

**___________________________________**

**Locus name OVA-BS4 cell lines**

**(parental and spheroids)**

**__________________________**

**AMEL** X

**D3S1358** 17

**D1S1656** 12-17.3

**D2S441** 11-14

**D10S1248** 16

**D13S317** 12

**Penta E**  10-17

**D16S539** 14

**D18S51** 13

**D2S1338** 17-25

**CSF1PO** 11

**Penta D** 9

**TH01** 9

**vWA**  16-19

**D21S11** 32.2

**D7S820** 9-10

**D5S818** 12

**TPOX** 9-11

**DYS391** -

**D8S1179** 13

**D12S391** 17.3-19

**D19S433** 14

**FGA**  23

**D22S1045** 16
